# *Centipeda minima*: A Review of Phytochemistry, Pharmacology, and Predictive Analysis on Quality Markers

**DOI:** 10.3390/molecules30204072

**Published:** 2025-10-13

**Authors:** Zhihong Shang, Yishuo Wang, Tianxin Zhu, Wenjing Niu, Tianai Lu, Rui Lou

**Affiliations:** 1 School of Pharmacy, Henan University of Chinese Medicine, Zhengzhou 450046, China; 2 Henan Engineering Technology Research Center for Integrated Technology of Traditional Chinese Medicine Production, Zhengzhou 450046, China

**Keywords:** *Centipeda minima*, chemical composition, pharmacological effects, quality marking objects, mechanism of action

## Abstract

As a traditional Chinese medicinal herb, *Centipeda minima* (L.) A. Braun & Asch is known for its effects in dispersing wind-cold, clearing nasal passages, and relieving coughs. Current research has identified various chemical constituents isolated from *C. minima*, including volatile oils, flavonoids, organic acids, and terpenoids. These compounds demonstrate multiple pharmacological activities such as anti-inflammatory, antioxidant, anti-allergic, and anti-tumour effects. This review summarizes the chemical constituents, pharmacological effects, and clinical applications of *C. minima*. Furthermore, based on the concept of Quality Markers (Q-Markers) in Chinese medicine, potential Q-Markers for *C. minima* are predicted and analyzed from five perspectives: botanical phylogeny, specificity of chemical composition, measurability of constituents, traditional efficacy, and medicinal properties. Compounds including brevilin A, arnicolide C, arnicolide D, and helenalin are proposed as candidate Q-Markers for *C. minima*, providing a scientific basis for elucidating its pharmacologically active substances and establishing quality evaluation criteria.

## 1. Introduction

*Centipeda minima* is the dried whole plant of *Centipeda minima* (L.) A. Br. et Aschers. (Asteraceae), also commonly known as chicken intestine herb, stone coriander, or ground coriander. It has a pungent taste and warm nature, acting on the lung meridian, and functions to disperse wind-cold, unblock the nasal passages, and relieve cough. It is clinically used for symptoms such as wind-cold headache, cough with phlegm, nasal congestion, and runny nose [1]. Modern studies have shown that it is rich in sesquiterpene lactones, flavonoids, triterpenoids, and volatile oils [2]. Among these, pseudoguaiacolide-type sesquiterpenes (e.g., brevilin A) have demonstrated significant anti-inflammatory and anti-allergic effects [3,4], showing potential applications in anti-allergy, anti-tumour, antioxidant, and neuroprotective activities. However, current research still has notable limitations: Firstly, the quality control method for *C. minima* specified in the *Pharmacopoeia of the People’s Republic of China* (2020 edition) includes only qualitative methods such as thin-layer chromatography and microscopic identification, with quantitation limited to a single compound (brevilin A), which is insufficient for comprehensively reflecting the herb’s quality. Secondly, the relationship between chemical constituents and medicinal efficacy has not been systematically studied, and critical quality attributes (CQAs) remain unclear. Thirdly, research on predicting quality markers (Q-Markers) based on a multi-dimensional ‘constituent-activity-mechanism’ network is still in its early stages, hindering the improvement of its quality standards.

The concept of Q-Markers offers a new paradigm for quality control of traditional Chinese medicine. It emphasizes the selection of quality indicators that are specific and traceable based on biosynthetic pathways, efficacy correlation, and component measurability, among other dimensions [5]. There is an urgent need to integrate chemomics, network pharmacology, and metabolomics to systematically analyze the ‘chemical composition–pharmacological activity–clinical application’ network and thereby construct a scientific and rational Q-Marker prediction model for *C. minima*.

This review retrieved relevant literature from databases including Web of Science (https://webofscience.clarivate.cn), PubMed (https://pubmed.ncbi.nlm.nih.gov), China National Knowledge Infrastructure (https://www.cnki.net), Wanfang Data (https://www.wanfangdata.com.cn), and Google Scholar (https://scholar.google.com), using keywords such as “*C. minima*”, “chemical composition”, and “pharmacological action”. After removing duplicate and irrelevant records, the collected literature and classical texts on Chinese medicine were summarized. Based on the Q-Marker theoretical framework, potential quality markers were explored from the perspectives of component specificity, bioactivity, deliverability, and measurability, aiming to provide a theoretical basis for improving the quality standards and supporting innovative drug development related to *C. minima*.

## 2. Chemical Composition

### 2.1. Volatile Oils

**Figure 1:**
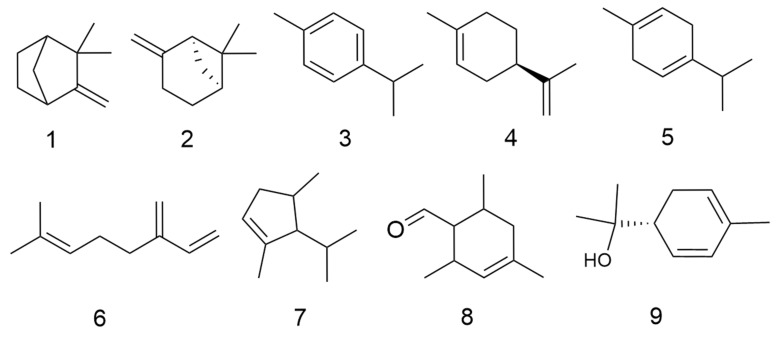

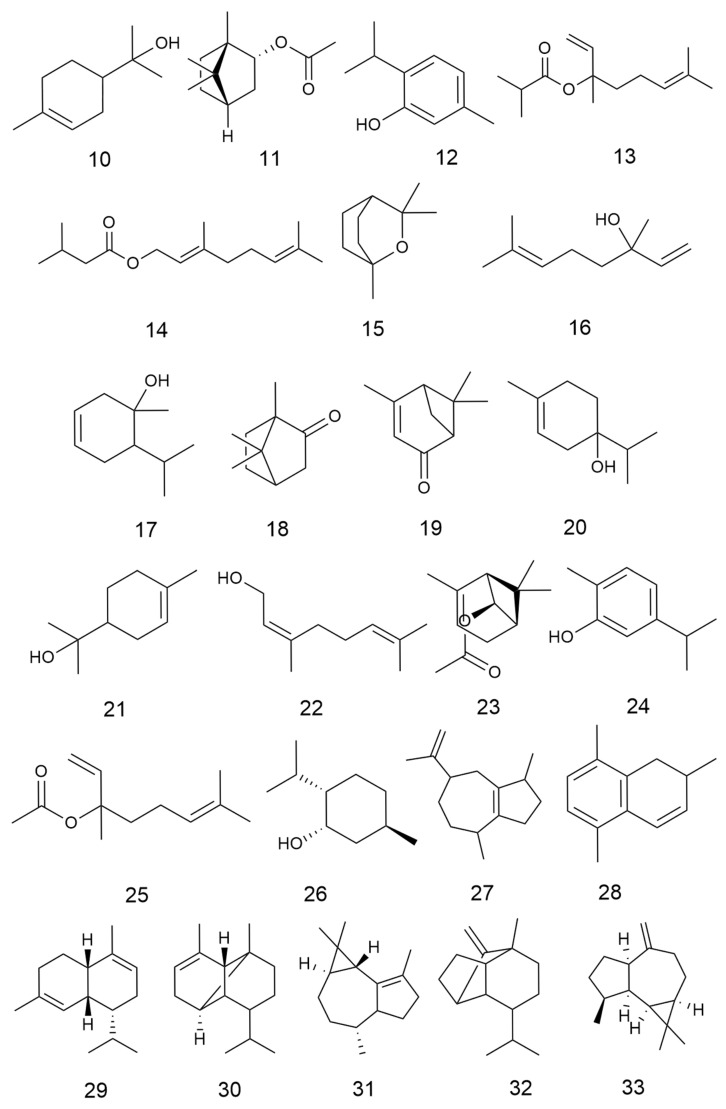

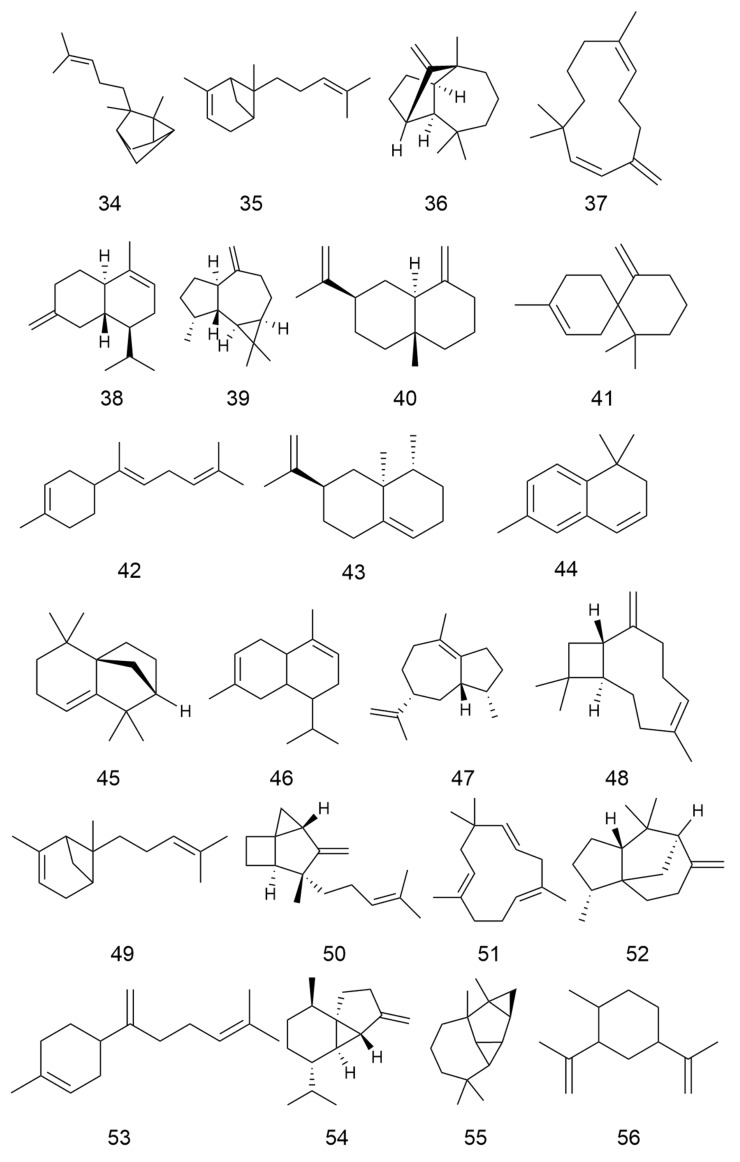

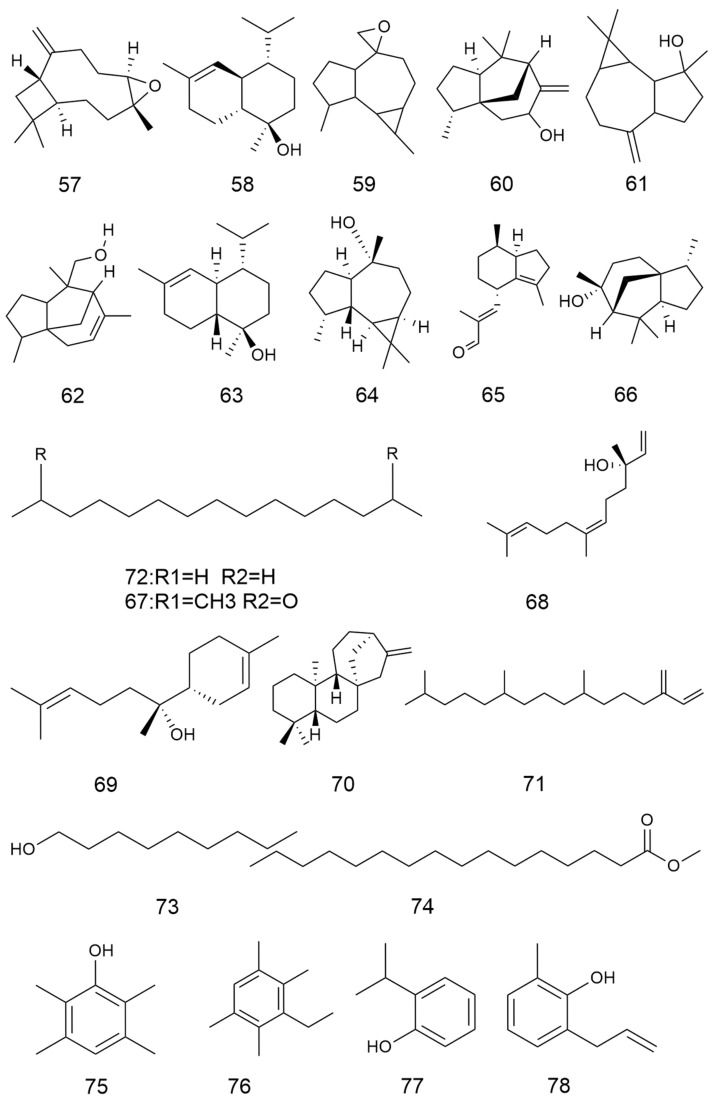

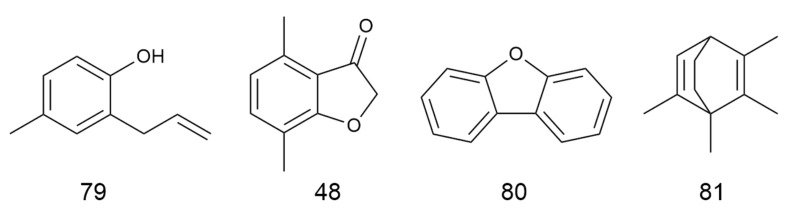
Volatile oils structure of *C. minima*. The numbers in the diagram correspond to those in Table 1.

*C. minima* is rich in volatile oil components with anti-inflammatory, antibacterial, antitumour, asthma and antiallergic activities [6]. Fan Su et al. [7] found that by extracting the volatile oils of *C. minima* and detecting them by GC-MS, the volatile oils of *C. minima* were mainly terpenes and esters, with the highest content of trans-chrysanthemum acetate, followed by aromatophilus, thymol, and β-stigmastanene, among others. A total of 86 volatile oil components have been identified. The specific composition is shown in Table 1. The compound structure is shown in Figure 1.

### 2.2. Terpenoids and Their Glycosides

**Figure 2:**
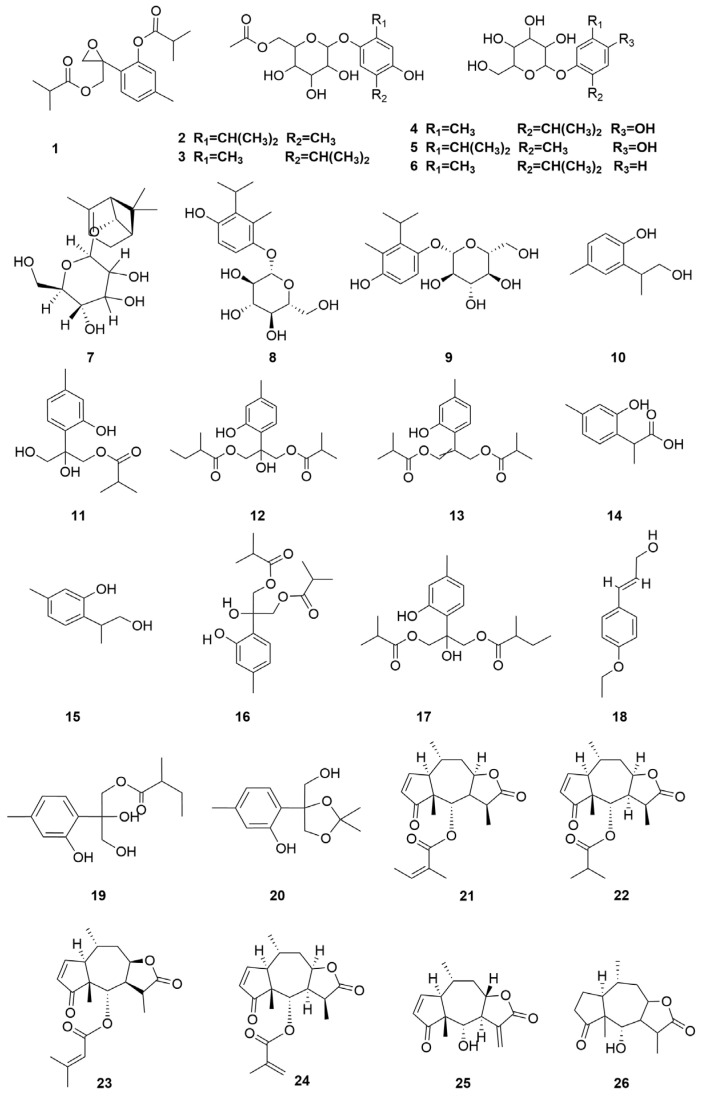

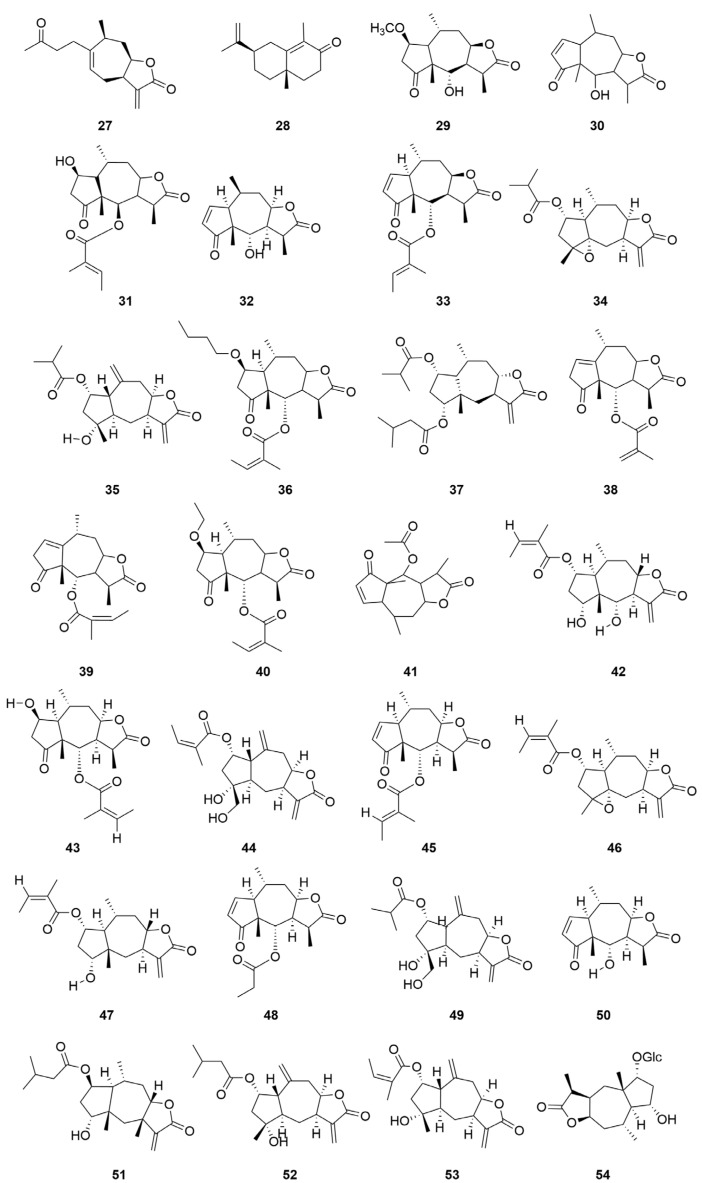

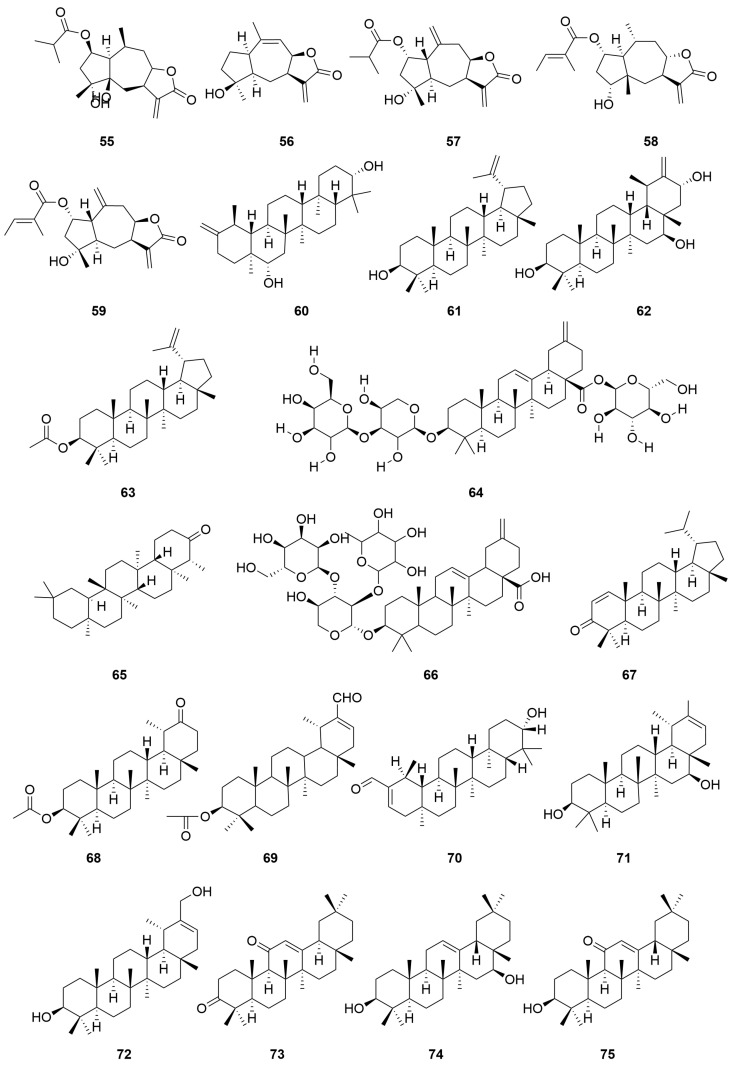

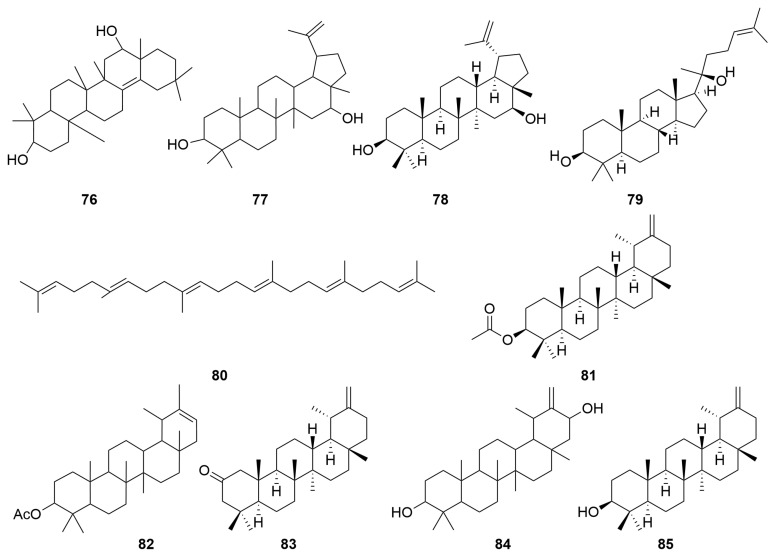
Terpenoids and their glycosides structure of *C. minima.* The numbers in the diagram correspond to those in Table 2.

*C. minima* contains a large number of terpenoids, of which guaiacolane sesquiterpene lactones are the main active constituents with biological activities such as anti-tumour, anti-inflammatory, anti-viral, anti-microbial, anti-malarial, anticancer, anti-diabetic, and analgesic [9], in addition to monoterpene glycosides, sesquiterpenes, and triterpene constituents, where triterpene compounds are mainly present in the form of pentacyclic triterpene saponins and their glycosides. Among these, the sesquiterpene lactones brevilin A, arnicolide D, and arnicolide C constitute the principal active constituents of *C. minima*. See Table 2 for details. The specific composition is shown in Table 2. The compound structure is shown in Figure 2.

### 2.3. Flavonoids and Their Glycosides

**Figure 3:**
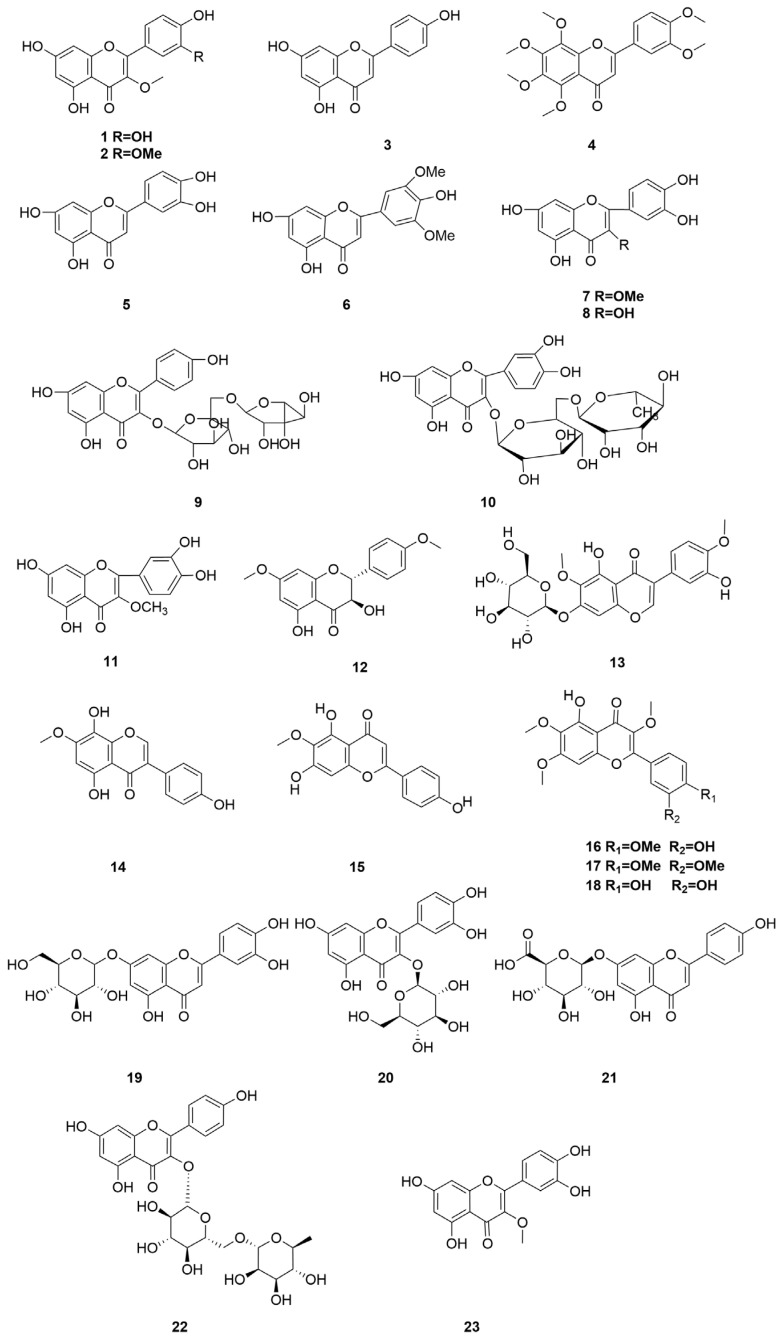
Terpenoids and their glycosides structure of *C. minima.* The numbers in the diagram correspond to those in Table 3.

*C. minima* is rich in flavonoids, with significant pharmacological activity, commonly used in clinical treatment of allergic rhinitis, and the anti-allergic effect of flavonoids in goose bugloss is slightly stronger than that of volatile oil components [32]. The specific composition is shown in Table 3. The compound structure is shown in Figure 3.

### 2.4. Sterols

**Figure 4:**
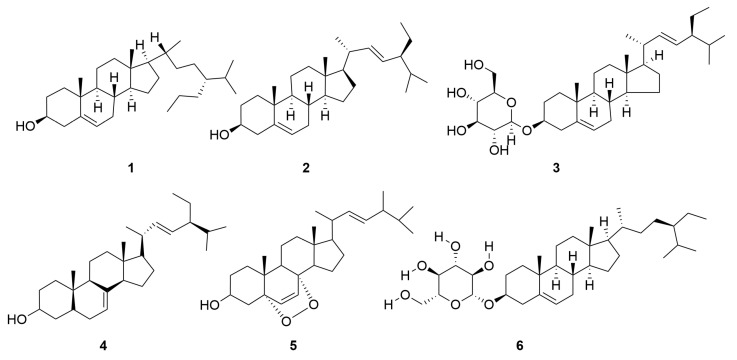
Sterols of *C. minima.* The numbers in the diagram correspond to those in Table 4.

A variety of sterol components have been extracted from *C. minima*, including β-sitosterol, stigmasterol and stigmasterol glucoside, totalling six. The specific composition is shown in Table 4. The compound structure is shown in Figure 4.

### 2.5. Organic Acids

**Figure 5:**
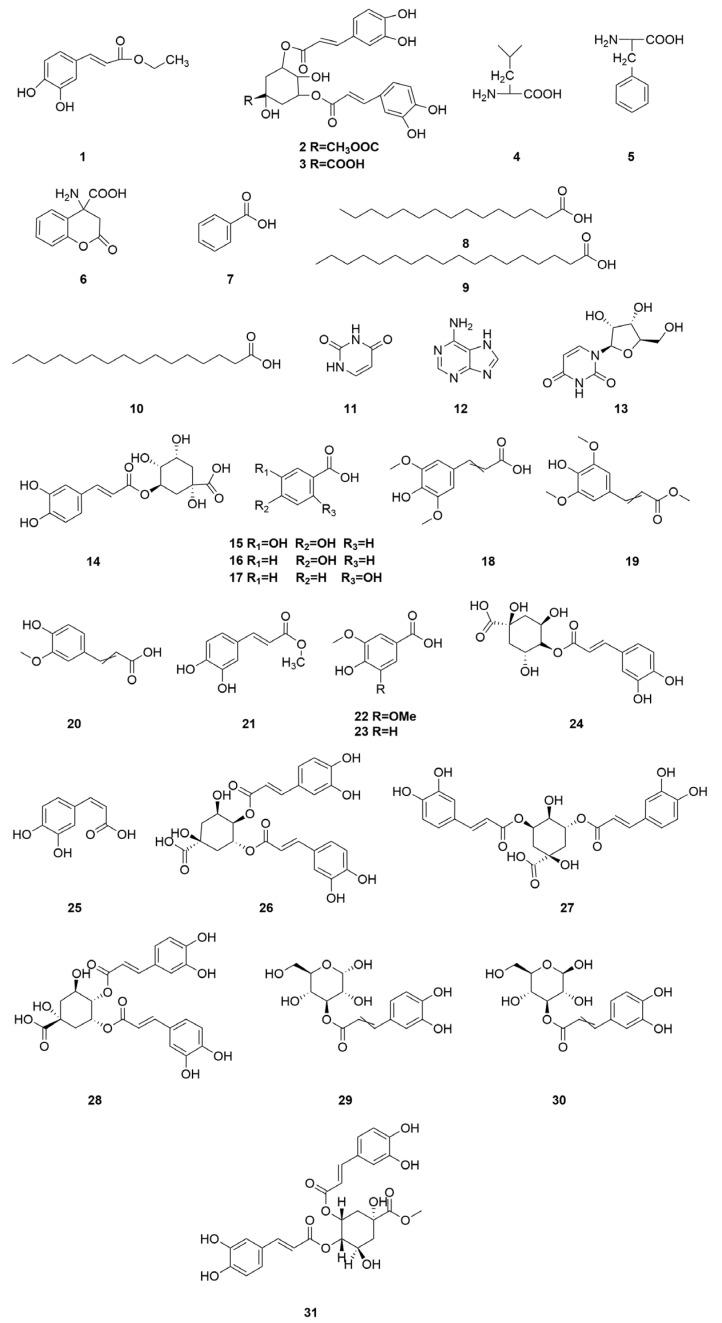
Organic acids structure of *C. minima.* The numbers in the diagram correspond to those in Table 5.

Organic acid components are important chemical constituents in *C. minima*, and the phenolic acid components have pharmacological activities such as antioxidant, antitumour, antimicrobial, antiviral, anti-inflammatory, antidepressant, and anxiolytic [38], but there are fewer studies on the activity of organic acids in *C. minima*. Nowadays, 30 organic acid components have been identified from *C. minima*. The specific composition is shown in Table 5. The compound structure is shown in Figure 5.

### 2.6. Other Chemical Compositions

**Figure 6:**
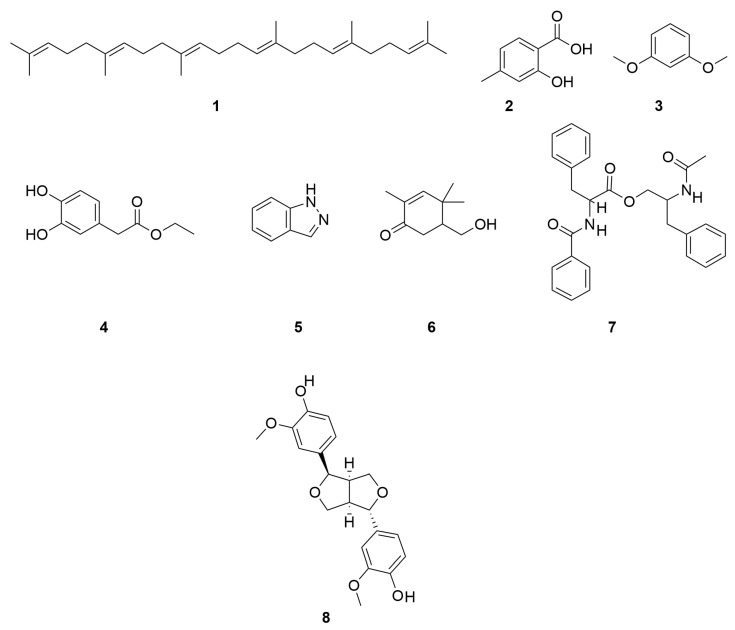
Other chemical compositions structure of *C. minima.*

Yang Liu et al. also identified Squalene (1), 2-hydroxy-4-methy-lbenzoic acid (2), 1,3-Dimethoxybenzene (3), ethyl-2-(3,4-dihydroxyphenyl)acetate (4), 1H-Indazole (5), 5-(hydroxymethyl)-2,4,4-trimethyl-2-cyclohexen-1-one (6) and patriscabratine (7) [16]. In addition to the above mentioned constituents, *C. minima* also contains epipinoresinol (8) and other constituents [33]. The compound structure is shown in Figure 6.

## 3. Pharmacological Effects

### 3.1. Anti-Allergy

**Figure 7:**
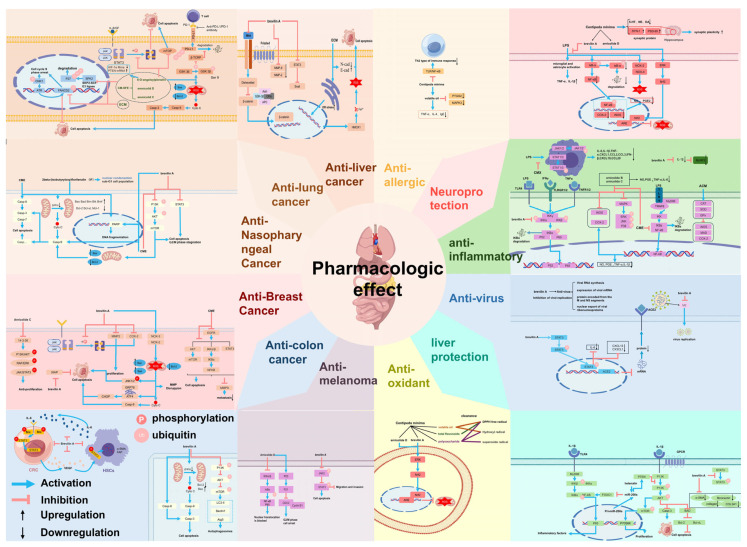
An overview of the pharmacological mechanism of action of the *C. minima.*

*C. minima* has potential components for anti-allergic effects and is mostly used clinically for the treatment of allergic rhinitis [25,41]. In the model group of allergic rhinitis, *C. minima* significantly improved the nasal epithelium with a large number of lysosomes, vacuoles of organelles, deformed nuclei, misplaced cells in the appropriate layer of connective tissue, damaged organelles, and infiltration of eosinophils and mast cells in the connective tissue [42]. It has been found that *C. minima* extract can effectively inhibit the TLR/NF-κΒ pathway involved in the cellular immune response and inflammatory response, which in turn attenuates the intensity of Th2-type immune response and treats allergic rhinitis [43]. Jia Yet al. [4] showed that *C. minima* volatile oil reduced the levels of TNF-α, IL-4, and IgE in the serum and significantly lowered the PTGS2 and MAPK3 protein levels, thereby treating allergic rhinitis. The specific mechanism of action is shown in Figure 7.

### 3.2. Anti-Tumour

#### 3.2.1. Anti-Lung Cancer

With 2.2 million new cases, lung cancer is the second most commonly diagnosed cancer and the leading type of cancer death in 2020 [44]. The most common types of lung cancer by tissue type are non-small cell lung cancer and small cell carcinoma. Fan XZet al. [45] found that the ethanol extract of *C. minima* could inhibit the Fanconi anemia pathway induced by DNA cross-linking agents by decreasing the expression of FANCD2 and mono-ubiquitylation, thus exerting synergistic anticancer effects with DNA cross-linking agents in the treatment of non-small cell lung cancer (NSCLC). Hu Qian et al. [46] found that alidiol, brevilin A, and helenalin-isovalerate exhibited a stronger, concentration-dependent inhibitory effect on lung cancer cells within the concentration range of 2.5–20 μmol/L, and the apoptosis-inducing effect of lung cancer cells was enhanced with the increase in the concentration of the components, and found that the mechanism of their action might be related to the inhibition of p-ERK, p-AKT, and NF-κB expression of p-ERK, p-AKT and NF-κB. Wang HC et al. [47] found that brevilin A, arnicolide D and arnicolide C in *C. minima* could exert anti-small cell lung cancer activity by inhibiting the Skp2/p27 signalling pathway, which induced cell cycle arrest and inhibited the migration of non-small cell lung cancer cells. brevilin A could play an anti-small cell lung cancer role by enhancing cancer cell killing activity through the enhancement of CD8 T cytotoxicity and the inhibition of GSK-3β-β-TRCP-mediated ubiquitylation and degradation de-inducing the expression of PD-L1 [48]. Wang R et al. [49] found that brevilin A significantly decreased HIF-1α mRNA expression and increased PTEN mRNA levels using network pharmacology and in vitro experimental validation, and hypothesized that *C. minima* could involve the HIF pathway in the treatment of lung cancer. Khan M et al. [50] found that brevilin A from *C. minima* inhibited proliferation and induced morphological changes in A549 and NCI-H1650 non-small cell lung cancer cells in a dose-dependent manner and increased ROS production and bax/bcl-2 ratio, while decreasing intracellular glutathione (GSH) and mitochondrial membrane potential, leading to induction of apoptosis. brevilin A also directly binds to STAT3, thereby inhibiting its activation and enhancing cytotoxicity. It has been found that brevilin A exerts its anticancer effect by generating ROS, and significantly inhibits the cell viability of lung cancer cells H1299 and A549 by inhibiting the Nrf2 antioxidant system, and does not have significant toxicity on non-cancer cells HB [51]. The specific mechanism of action is shown in Figure 7.

#### 3.2.2. Anti-Nasopharyngeal Cancer

*C. minima* is also effective against nasopharyngeal carcinoma. Lin Yusan et al. [21]. tested the inhibitory effect of compounds on tumour cell proliferation by MTT assay, and found that brevilin A, Arnicolide C, and Arnicolide D had a more obvious inhibitory effect on nasopharyngeal carcinoma cells CNE-1 and CNE-2, and their IC50 values were all less than 10 μmol/L-1, which was close to that of the positive drug, cisplatin. Su M [26] showed significant dose- and time-dependent inhibition of human nasopharyngeal carcinoma epithelial (CNE) cell growth by 2beta-(Isobutyryloxy)florilenalin (IF) isolated from *C. minima*, inducing apoptosis of CNE cells, resulting in accumulation of sub-G1 cell population, DNA fragmentation and nuclear condensation, Caspase-3 activation and PARP cleavage, which in turn leads to caspase cleavage and cell death. It was found that goosefoot extract inhibited PI3K-Akt-mTOR signalling and cell proliferation in nasopharyngeal carcinoma CNE-1 cells [52]. Brevilin A and Arnicolide D can activate the Caspase signalling pathway by regulating cell cycle proteins, inhibit the PI3K/AKT/mTOR and STAT3 signalling pathways, and thus inhibit the nasopharyngeal carcinoma cell growth [53,54]. The specific mechanism of action is shown in Figure 7.

#### 3.2.3. Anti-Liver Cancer

Liver cancer is the fifth most common cancer and the fourth leading cause of cancer-related deaths worldwide. There are two main types of primary liver cancer—hepatocellular carcinoma (HCC) and intrahepatic cholangiocarcinoma (ICC)—and approximately 70% of colorectal cancer patients develop secondary liver cancer [55]. Wei J et al. found that ethanolic extract of goosefoot significantly inhibited the G2/M cell cycle, up-regulated HMOX1 expression through ER stress, leading to Fe^2+^, ROS accumulation, and apoptosis, and inhibited invasion and cell migration of hepatocellular carcinoma through down-regulation of N-cad and up-regulation of E-cad [56]. Qin Y et al. found that brevilin A-treated HepG2 and SMMC-7221 cells showed a significant increase in apoptosis and a decrease in the expression levels of MMP-2 and MMP-9 [57]. In addition, brevilin A inhibited the Stat3/Snail and Wnt/β-catenin signalling pathways in HCC cells, which led to the inhibition of hepatocellular carcinoma. The specific mechanism of action is shown in Figure 7.

#### 3.2.4. Anti-Breast Cancer

Since 2020, breast cancer has surpassed lung cancer as the most prevalent malignancy in women, and it is one of the leading causes of cancer-related deaths globally [58]. Liu Z et al. [59] found that Arnicolide C in *C. minima* induced cell cycle arrest and increased apoptosis, and hypothesized that it exerts anti-breast cancer effects by affecting the level of 14-3-3θ protein expression Wen W et al. [60] found that arnicolide D inhibited oxidative stress-induced breast cancer cell growth and invasion through apoptosis and iron death. Lee MM et al. [61] found that the total extract of *C. minima* significantly reduced cell viability and proliferation and induced a significant reduction in cell viability and proliferation in a dose- and time-dependent manner in MDA-MB-231 xenograft mice. They found that in MDA-MB-231 xenograft mice, *C. minima* extract could significantly reduce cell viability and proliferation in a dose-dependent and time-dependent manner, induce apoptosis, and inhibit cancer cell migration and invasion, and exert its anti-triple-negative breast cancer effects through multi-targets and multi-pathways, such as the EGFR, PI3K/AKT/mTOR, NF-κB, and STAT3 pathways, and cause a decrease in MMP-9 activity and metastasis inhibition in vitro, without any side-effects. It was also found that brevilin A induced mitotic G2/M block, ROS-dependent apoptosis, endoplasmic reticulum (ER) stress, mitochondrial dysfunction, and inhibited STAT3 activation in MCF-7 cells, thereby inhibiting MCF-7 breast cancer cell activity [62]. The specific mechanism of action is shown in Figure 7.

#### 3.2.5. Anti-Colon Cancer

Colorectal cancer (CRC) is one of the most common cancers worldwide and its incidence is increasing every year, with high-fat, low-fibre diets and colonic inflammation as possible contributing factors [63]. Brevilin A was found to target the VEGF-IL6-STAT3 axis in colorectal cancer-hepatic stellate cells (CRC-HSCs) interactions, resulting in significant inhibition of colorectal liver metastasis and further cancer progression in vitro and in vivo [64]. You P [65] found that brevilin A could also induce apoptosis and autophagy in CT26 colon adenocarcinoma cells via the mitochondrial pathway and PI3K/AKT/mTOR inactivation by increasing the ROS level, decreasing the mitochondrial membrane potential (MMP), and inducing apoptosis in CT26 cells in a dose-dependent manner. The apoptosis induced by brevilin A was higher than that induced by adriamycin at the same dose, and the up-regulation of cleaved-caspase-8, cleaved-caspase-9, and cleaved-caspase-3, the increase in Bax protein expression, and the decrease in Bcl-2 were observed after brevilin A treatment. Further studies showed that brevilin A inhibited the phosphorylation of PI3K, AKT and mTOR, and promoted the expression of autophagy-related proteins LC3-II, Beclin1 and Atg5, resulting in the formation of autophagosomes, whereas 3-methyladenine (3-MA), a type III PI3K inhibitor, inhibited the brevilin A-induced autophagosome formation. The specific mechanism of action is shown in Figure 7.

#### 3.2.6. Anti-Melanoma

Melanoma is an extremely aggressive cancer prone to metastasis, primarily affecting the skin and mucous membranes, with a steadily increasing global patient population [66]. *C. minima* exhibits certain therapeutic efficacy for this disease. Research by Su T et al. [67] revealed that brevilin A reduces cell viability, induces apoptosis, inhibits migration and invasion, and lowers the protein levels of phosphorylated JAK2 (Y1007/1008) and phosphorylated STAT3 (Tyr705). Concurrently, it suppresses STAT3 expression, thereby exerting anti-melanoma effects. Zhu P et al. [68] found that Arnicolide D also exerted an antimelanoma effect, mainly by decreasing cell viability, inducing G2/M cell cycle arrest and apoptosis, and increasing the levels of the cell cycle regulatory proteins p53 and p21 in melanoma cells. Arnicolide D also exerts antimelanoma effects by reducing cell viability, inducing G2/M cell cycle arrest and apoptosis, increasing the levels of p53 and p21, and decreasing the levels of the G2/M checkpoint protein Cdc2 and the cell cycle protein B1, through the mechanisms of inhibiting the activity of IKKα/β, the degradation of IκBα, and the phosphorylation and expression of NF-κB p65 in melanoma cells. The specific mechanism of action is shown in Figure 7.

#### 3.2.7. Others

In addition to the above cancers, *C. minima* can also be used in the treatment of prostate cancer, osteosarcoma, gastric cancer, etc. You P et al. [69] found that Brevilin A in *C. minima* inhibited prostate cancer cell proliferation, migration and invasion, suppressed the expression of lncRNA H19 and E2F3, and enhanced the level of miR-194. It regulates prostate cancer cells through the lncRNA H19/miR-194/E2F3 axis. Chen Z et al. [70] also found that Arnicolide D in *C. minima* inhibited the activation of the PI3K/Akt/mTOR pathway in osteosarcoma cells, which has the effect of inhibiting osteosarcoma cell proliferation and inducing apoptosis. Lee D et al. [71] found that Brevilin A induced apoptosis by up-regulating the expression of cleaved caspase-8 and cleaved caspase-3, reducing the ratio of Bax to Bcl-2, and achieving anti-gastric cancer effects. Wang J et al. [72] found that Brevilin A induced oxidative stress and mitochondrial dysfunction, thereby inhibiting the proliferation of U87 glioblastoma cells and inducing apoptotic cells in a dose-dependent manner and inducing severe morphological changes and apoptotic cell death. Brevilin A also induced apoptosis in human leukemia HL60 cells by stimulating reactive oxygen species (ROS) production, lowering the mitochondrial transmembrane potential (DeltaPsim), and activating caspase-3/7; inhibited the activation of NF-kappaB, regulating the Bcl-2 gene family expression and disruption of mitochondrial function-induced apoptosis; and even inhibited solid cancer growth in the Lewis lung cancer xenograft model [73].

### 3.3. Anti-Depressant (Neuroprotective)

Depression is a very threatening disease to mental health, and *C. minima*, a traditional Chinese medicine, has some neuroprotective effects. Dou Shi-Ying [74] and others found that *C. minima* improved serum monoamine neurotransmitter contents such as 5-HT, NE and DA in rifampicin-induced depressed rats, improved hippocampal tissue morphology damage and increased the content of synaptic proteins SYN-1 and PSD-95 in the hippocampus, and speculated that its mechanism of action might be related to the increase in serum monoamine neurotransmitter contents and the improvement of hippocampal. Zhou, Y.L. [75] revealed that Brevilin A, a natural sesquiterpene lactone from *C. minima*, dose-dependently inhibited LPS-induced microglia activation, decreased the levels of inflammatory factors such as TNF-α and IL-1β, inhibited the nuclear translocation of NF-κB and the expression of COX-2/iNOS, and reduced the levels of NO and PGE2 production, which can effectively alleviate the inflammatory response of brain tissue, inhibit glial cell activation, and also protect neuronal and synaptic structures, and thus exert neuroprotective effects against lipopolysaccharide-induced neuroinflammation in vitro and in vivo. High levels of ROS and aberrant redox changes significantly induced neuronal death and enhanced the pathogenesis of neurodegenerative diseases, whereas *C. minima* ethanolic extract possessed antioxidant activity and was able to protect neuronal cells from oxidative stress-induced damage through activation of ERK/Nrf2 signalling pathway, and significantly improved learning and memory ability in mice. Brevilin A and arnicolide D are the main components responsible for activating the Nrf2 signalling pathway and inhibiting ROS production, thus exerting neuroprotective effects [76,77]. The specific mechanism of action is shown in Figure 7.

### 3.4. Antioxidant

Endogenous reactive oxygen species (ROS) play an important role in the human body, and oxidative stress is a phenomenon caused by an imbalance between the production and accumulation of oxygen-responsive substances (ROS) in cells and tissues and the ability of the biological system to detoxify these reaction products, which leads to oxidative damage, disrupts membrane integrity or leads to cell death, and induces a series of diseases in the body [78]. Wang, Y.J. et al. [76] found that the ethanolic extract of *C. minima* exhibited significant antioxidant activity and revealed its mechanism of action, isolating brevilin A and arnicolide D as the active compounds responsible for the activation of the Nrf2 signalling pathway and the inhibition of ROS production. The total flavonoids of *C. minima* showed strong scavenging effects on DPPH radicals [79], and the volatile oil was found to have antioxidant effects on *C. minima* through the scavenging effect experiments on DPPH radicals and hydroxyl radicals: essential oil > carvacrol > thymol [7].Yang, G. et al. [80] found that *C. minima* polysaccharides could also exhibit good antioxidant activity with potential to scavenge DPPH radicals, hydroxyl radicals and superoxide radicals. The specific mechanism of action is shown in Figure 7.

### 3.5. Antibacterial and Anti-Inflammatory

*C. minima* essential oil inhibits the growth of *S. aureus* and inhibits biofilm formation by altering cell membrane permeability and interfering with metabolic activities in bacteria in vivo [7]. *C. minima* possesses three antimicrobial sesquiterpene lactone components 6-O-methylacrylylplenolin, 6-O-isobutyroylplenolin, and 6-O-angeloylplenolin with activity against Bacillus subtilis and Staphylococcus aureus, with 6-O-isobutyroylplenolin being the most active [81].

Total extract of *C. minima* showed inhibitory effects on NF-κB, STAT3 and MAPK signalling in macrophages and CCL8 expression in activated macrophages and ameliorated inflammatory bowel disease [82]. The aqueous extract of *C. minima* showed anti-inflammatory activity by decreasing the levels of malondialdehyde (MDA), nitric oxide synthase (iNOS) and cyclooxygenase-2 (COX-2) by increasing the activities of catalase (CAT), superoxide dismutase (SOD), and glutathione peroxidase (GPx) [83]. *C. minima* ethanolic extract protected HT22 neuronal cells from inflammatory damage by reducing the production of pro-inflammatory mediators tumour necrosis factor-α (TNF-α) and interleukin-1β (IL-1β), inhibiting the phosphorylation of NF-κB, and decreasing the expression of COX2, iNOS, NOX2, and NOX4 in hippocampal tissues, and found that LPS-induced microglia activation was significantly attenuated in the hippocampus, whereas a high dose of *C. minima* ethanol extract possessed stronger anti-inflammatory activity [84]. Penghui Xue et al. [29] determined the inhibitory activity of *C. minima* extract on lipopolysaccharide (LPS)-induced release of the inflammatory mediator NO from mouse macrophages (RAW264.7) using Griess method, and found that 3β-acetoxytaraxaster-20-en-30-al, 3β,16β-hydroxy-lupinidiol, 16β-hydroxy-lupin-20(29)-en-3-one, and garcinielliptone Q had significant anti-inflammatory activity. *C. minima* extract significantly inhibited JAK1/2 and STAT1/3 phosphorylation in macrophages and inflammatory response in keratin-forming cells [85]. *C. minima* arnicolide B and arnicolide C in *C. minima* not only inhibited the production of inflammatory mediators NO, PGE2, TNF-α and IL-6, but also down-regulated the high expression of inflammatory proteins iNOS and COX-2. In addition, they can inhibit the phosphorylation of ERK, JNK, and p38 proteins in the MAPK signalling pathway, thus exerting anti-inflammatory effects [86]. Liu L et al. [87] found that Brevilin A effectively reduced LPS-induced inflammatory lung injury by targeting IKKα/β and inhibiting NF-κB signalling. Xue PH et al. [88] identified three anti-inflammatory components in *C. minima*, centiplide A, centiplide H, and helenalin-isovalerate, which exhibited significant inhibitory activity on nitric oxide production in a lipopolysaccharide-activated macrophage cell line of RAW 264.7 mice. Qin Qin et al. [3] found that *C. minima* could effectively reduce LPS-induced inflammatory lung injury by targeting IKKα/β to inhibit NF-κB signalling. found that Brevilin A in *C. minima* significantly reduced IL-1β secretion to inhibit NLRP3 inflammatory vesicles. The specific mechanism of action is shown in Figure 7.

### 3.6. Hepatoprotective Effect

Sesquiterpene lactones from *C. minima* have hepatoprotective activity. Bin Fang found that Helenalin significantly inhibited the proliferation, migration and colony formation of Hepatic stellate cells (HSCs), induced apoptosis of HSCs, alleviated inflammation, regulated the balance of MMPs/TIMPs, and reduced the synthesis of collagen in activated HSCs, and the mechanism of Helenalin may be related to the up-regulation of miR-200a and the inhibition of NF-κB and PI3K/Akt signalling pathways mediated by miR-200a [89]. Brevilin A significantly inhibited STAT3 phosphorylation, which was increased by TGF-β treatment, and reduced the expression levels of fibronectin and connective tissue growth factor, which in turn exerted an antifibrotic effect in liver fibrosis [90]. The specific mechanism of action is shown in Figure 7.

### 3.7. Antiviral

Brevilin A from *C. minima* is a potent Angiotensin-converting enzyme 2 (ACE2) inhibitory component that also inhibits the entry of SARS-CoV-2 S-protein pseudoviruses into the target cells, thereby protecting lung epithelial cells from SARS-CoV-2 infection [91]. Zhang, X.L. et al. [92] tested the anti-influenza virus A/Puerto Rico/8/34 HIN1 (PR8) activity of nine sesquiterpene lactones using the Cellular Pathogenic Effect (CPE) Reduction and Cell Counting Kit 8 (CCK8) assay and found that brevilin A showed the strongest anti-PR8 activity with IC50 values of 0.01 and 0.01, respectively, and that brevilin A affected PR8 replication through down-regulation of the expression of viral M2 protein. It was found that brevilin A exhibited the most potent anti-PR8 activity by down-regulating the expression of the viral M2 protein, affecting the intracellular replication of PR8, and the IC50 value was much lower than that of the positive control, ribavirin. and H9N2) [93]. The specific mechanism of action is shown in Figure 7.

### 3.8. Others

In addition to this, *C. minima* has pharmacological effects such as asthma-calming, hair growth, anti-angiogenesis, and anti-mitosis effects.

Chen Qiang et al. [94] used chloroacetylcholine and histamine phosphate to create an asthma model; *C. minima* volatile oil can significantly extend the effect of guinea pig-induced asthma latency after oral administration, and through the contraction of isolated guinea pig tracheal smooth muscle spiral strip contraction experiments, they found that the volatile oil of *C. minima* can antagonize the effect of histamine phosphate on the H1 receptor to produce bronchial smooth muscle contraction and exert an asthmatic effect. 

A study found that *C. minima* can enhance the expression of vascular endothelial growth factor (Vegfa) and insulin-like growth factor (Igf1), inhibit the expression of transforming growth factor β 1 (Tgfb1), promote the development of hair follicles, and then promote hair growth in C57BL/6 mice [95]. *C. minima* extract has also been found to promote hair growth and growth factor secretion through the Wnt/β-catenin, ERK and JNK signalling pathways, and its compounds have been shown to have high bioavailability and gastrointestinal absorption [96].

Brevilin A in *C. minima* activates p38/PMK-1 in the gut for innate immune responses, increases resistance to oxidative stress and prolongs lifespan through the p38 MAPK pathway, and enhances resistance to pathogens by reducing bacteria in the gut [97].

*C. minima* and its Brevilin A constituent have been shown to possess antiangiogenic activity [98]. The (Z)-3,5,4′-Trimethoxystilbene in *C. minima* exerts antimitotic activity by inhibiting microtubule protein polymerisation in a dose-dependent manner [99].

## 4. Security Evaluation

Oral administration of *C. minima* exhibits gastrointestinal irritancy. Within a certain period after ingestion, symptoms such as pharyngeal, esophageal, and gastric burning sensations, nausea, vomiting, gastralgia, abdominal pain (sometimes severe), and even back pain and generalized myalgia have been reported in some patients [100].

Using the screening criteria of oral bioavailability (OB) ≥ 30% and drug-likeness (DL) ≥ 0.18, active constituents of *C. minima* were retrieved from the TCMSP platform (https://www.tcmsp-e.com/tcmsp.php) After standardization using the Uniprot database, 197 potential target gene standard abbreviations were obtained. Disease target genes were retrieved and screened using the keywords “gastric irritation” and “gastric mucosal injury” from the GeneCards (http://www.genecards.org/) and OMIM (http://omim.org/) databases. After merging and deduplication, 7110 disease targets were identified. Venn diagram analysis of the constituents and gastric irritation targets yielded 179 common targets, providing corroborating evidence for the documented irritancy.

However, clinically, only a low incidence of adverse reactions has been observed. Furthermore, no documented toxicity exists in historical records of Traditional Chinese Medicine (TCM), indicating that *C. minima* is a relatively mild and safe medicinal herb with significant therapeutic value, and it is widely used in TCM practice. Pharmacological experiments revealed that oral administration in mice showed no signs of weight loss or hepatorenal toxicity, as evidenced by the levels of serum transaminases, alanine aminotransferase, creatinine, and blood urea nitrogen concentrations [45,101], demonstrating a favourable safety profile. Current research on *C. minima* is limited. To better assess its safety and potential side effects, further systematic toxicological studies are warranted in the future.

## 5. Clinical Applications

*C. minima* is widely used in the clinic, mostly as a single medicine or in compound formulas, and is commonly used for the treatment of allergic rhinitis, whooping cough, stones, headache, malaria, and hookworm tail pass infections [102]. *C. minima* is also commonly used in combination with *Magnolia*, *Xanthium sibiricum Patr.* and *Asari Radix* et Rhizoma and other drugs to treat allergic rhinitis [103]. Various compounded formulations have been developed, such as Nasonex Nasal Drops [104], Resting Cough and Anti-inflammatory Bulk [105], *C. minima*-formulated granules [106], Kelisha capsule [107], and Thermo Kepin injection [108]. One of the allergic rhinitis nasal drops (ARND) not only inhibits viral infections and disrupts the affinity between ACE2 and the spiking protein (Delta), but also attenuates the inflammatory response after infection, which may lead to a better prognosis and lower risk of pulmonary fibrosis [109]. Kelisha capsule, by inhibiting the activity of the enzyme ATP synthase in Escherichia coli, reduces the ATP levels, as well as disrupting the cell wall and cell membrane of E. coli, leading to cytoplasmic leakage and bacterial death to exert its antimicrobial effect [107].

## 6. Quality Marker Prediction

### 6.1. Predictive Analysis of Quality Markers Based on Plant Affinities and Chemical Specificity

*C. minima* belongs to the genus *Centipeda Lour.* in the subtribe *Chrysantheminae O.Hoffm.*, tribe *Anthemideae Cass.*, subfamily *Carduoideae Kitam* of the *Asteraceae family* (also known as *Compositae*). The Asteraceae family comprises approximately 1000 genera and 25,000–30,000 species worldwide, with relatively fewer representatives in tropical regions. In China, this family encompasses over 200 genera and more than 2000 species distributed across the country, including approximately 155 genera and 778 species with medicinal value [110]. The genus *Centipeda* contains six species globally, distributed across Asia, Oceania, and South America, with *C. minima* being the sole representative species in China. Plants in the Asteraceae family predominantly contain bioactive compounds such as terpenoids, flavonoids, and phenylpropanoids, with sesquiterpene lactones and flavonoids being particularly ubiquitous [111]. The phytochemical profile of Centipeda species aligns with this pattern, containing terpenoids, flavonoids, phenylpropanoids, volatile oils, sterols, and organic acids. Within the subtribe *Chrysantheminae*, medicinal genera such as *Artemisia* and *Chrysanthemum* are noteworthy. Artemisia species are characterized by essential oils, flavonoids, and organic acids [112], while *Chrysanthemum* species primarily produce terpenoids, flavonoids, polyacetylenes, phenylpropanoids, alkaloids, and steroids [113]. Comparative analysis reveals that *C. minima* contains distinctive sesquiterpene lactones, including brevilin A, arnicolide C, and arnicolide D, which are absent in other genera within the family. These unique compounds—brevilin A, arnicolide C, and arnicolide D—may therefore serve as potential Quality Markers (Q-Markers) for the authentication and quality control of *C. minima*.

### 6.2. Predictive Analysis of Quality Markers Based on Correlation Between Composition and Efficacy

*C. minima* is characterized as pungent in flavour, warm in nature, and associated with the lung meridian. It is traditionally used to address wind-cold headaches, cough with excessive phlegm, nasal congestion, and sinusitis with rhinorrhea [1]. The pungent flavour in traditional Chinese medicine (TCM) theory is described as “capable of dispersing and moving,” reflecting its ability to promote circulation and resolve stagnation. Pungent herbs typically contain volatile oils, glycosides, terpenoids, and alkaloids [114]. Warm-natured herbs are often associated with volatile oils, esters, organic acids, carbohydrates, inorganic compounds, alkaloids, and flavonoids, which collectively form their bioactive foundation [115]. Notably, lung meridian-targeting herbs frequently contain volatile oils, glycosides, flavonoids, and terpenoids, exhibiting pharmacological activities such as anti-inflammatory, antimicrobial, and antitumor effects [116]. *C. minima* predominantly contains volatile oils, flavonoids, terpenoids, and organic acids [117], making these classes of compounds potential quality markers (Q-Markers) for this herb.

Brevilin A, arnicolide C, arnicolide D, and 2β-(isobutyryloxy)florilenalin (IF) demonstrate significant anti-tumour activity, while 6-O-angeloylplenolin and arnicolide D exhibit neuroprotective effects. Anti-inflammatory activity is observed in compounds such as 3β-acetoxytaraxaster-20-en-30-al, 3β,16β-dihydroxylup-20(29)-en-3-one, 16β-hydroxylup-20(29)-en-3-one, brevilin A, centiplide A, centiplide H, helenalin-isovalerate, garcinielliptone Q, arnicolide B, and arnicolide C. Antimicrobial activity is attributed to 6-O-methylacrylylplenolin, 6-O-isobutyroylplenolin, and brevilin A. Additionally, helenalin and brevilin A display hepatoprotective properties, while brevilin A exhibits antiviral activity.

In summary, Brevilin A, 2beta-(Isobutyryloxy)florilenalin (IF), 3beta-acetoxytaraxaster-20-en-30-al, 3beta,16beta-hydroxy-lupine diol, 16beta-hydroxy-lupin-20(29)-en-3-one, centiplide A, centiplide H, helenalin-isovalerate, garcinielliptoneQ, arnicolide B and arnicolide C, 6-O-methylacrylylplenolin, 6-O- isobutyroylplenolin, Helenalin can be used as a reference for the selection of *C. minima* Q-Markers.

### 6.3. Predictive Analysis of Quality Markers Based on Chemical Composition Measurability

The chemical components in *C. minima* are abundant, and the substances that are stable and can be quantitatively determined are clearly defined in order to better establish a quality control evaluation system, and the selected chemical components should have a certain content and be able to have a content determination method that meets the exclusivity [118]. Zan Ke et al. [119] employed high-performance liquid chromatography (HPLC) to establish a characteristic fingerprint of the medicinal herb *Achillea millefolium*, whilst simultaneously determining the content of chlorogenic acid, cryptoclorogenic acid, caffeic acid, rutin, isochlorogenic acid B, isochlorogenic acid A, and isochlorogenic acid C. Ren Haiqin et al. [120] established a method for the simultaneous measurement of arnicolide D, arnicolide C, minimolide F, microhelenin C and brevilin A by HPLC. Chan CO et al. [19] established a method for the simultaneous measurement of arnicolide D, arnicolide C, minimolide F, microhelenin C, and brevilin A by ultra performance liquid chromatography coupled with triple quadrupole mass spectrometry (UPLC-QQQ-MS) and UPLC-High Resolution Orbitrap Mass Spectrometry (UPLC-Orbitrap-MS) initially identified 15 sesquiterpene lactones in the methanolic extract of *C. minima*, and found that Brevilin A and arnicolide D were the major sesquiterpene lactone components in *C. minima*. Chan CO simultaneously determined chlorogenic acid, cryptoclorogenic acid, caffeic acid, rutin, isochlorogenic acid B, kaempferol-3-O-rutinoside, isochlorogenic acid A, isochlorogenic acid C, 3-methoxyquercetin, brevilin A, arnicolide D, arnicolide C [20]. The contents of these acids were determined by HPLC-DAD. Thin-layer chromatography was used to identify *C. minima* oil, and gas chromatography internal standard method was used to determine the content of chrysanthenyl acetate and thymol in *C. minima* oil [121]. Chen Wei et al. [122] quantified 16 batches of *C. minima* from 7 provinces by HPLC and found that chlorogenic acid, isochlorogenic acid A, isochlorogenic acid C, quercetin, kaempferol, 3-methoxyquercetin, arnicolide D, arnicolide C, microhelenin C, short brevilin A, lupinol, β-sitosterol, and taraxasterol varied considerably among provenances.

In summary, chlorogenic acid, quercetin, brevilin A, rutin, isochlorogenic acid B, isochlorogenic acid A and isochlorogenic acid C, arnicolide D, arnicolide C, minimolide F, microhelenin C, cryptochlorogenic acid, caffeic acid, nicotiflorin, 3-methoxyquercetin, chrysanthenyl acetate, and thymol may inform the Q-Marker selection of *C. minima*.

### 6.4. Predictive Analysis of Quality Markers Based on Incoming Blood Components

An in vivo pharmacokinetic study of rutin and 3-O-methyl quercetin in the alcoholic extract of *C. minima* revealed that the rutin prototype was detected in plasma, the liver at 1 h, the kidney at 4 h, brain tissue at 15 min–8 h, and urine at 0–24 h in rats that were orally administered with the extract, and stated that rutin could cross the blood–brain barrier (BBB) in rats and 3-O-methylquercetin was detected in bile [123]. Intravenous and intraperitoneal injection of brevilin A and detection of blood by LC-MS revealed that in intraperitoneally injected rats, the bioavailability of brevilin A was 92.5%, indicating that its therapeutic concentration could be achieved by intravenous and intraperitoneal injection of brevilin A [124].

In conclusion, rutin, 3-O-methylquercetin, and brevilin A can be used as blood-entry components with good pharmacokinetic profiles, and can be used as potential choices for the Q-Marker of *C. minima*.

### 6.5. Predictive Analysis of Quality Markers Based on Correlation of Ingredients with Modern Clinical Use

Currently *C. minima* is available in the market mostly in the form of formulated granules, sprays, capsules, dispersions, and injections, which are commonly used for the treatment of symptoms such as rhinitis, fever, and cough, etc. *C. minima* formulated granules contain caffeic acid, isochlorogenic acid A, isochlorogenic acid C, isochlorogenic acid B, cryptochlorogenic acid, and neochlorogenic acid [106]. Nasonex spray has been found to contain components such as chlorogenic acid, rutin, isochlorogenic acid A, quercetin, and kaempferol [125]. Nasonex Spray has been found to contain components such as chrysanthenyl acetate [126].

In summary, caffeic acid, isochlorogenic acid A, isochlorogenic acid C, isochlorogenic acid B, cryptochlorogenic acid, and neochlorogenic acid, chrysanthemum acetate, chlorogenic acid, rutin, isochlorogenic acid A, quercetin, and kaempferol may be potential choices for Q-Marker of *C. minima*.

### 6.6. Summary

Based on the five principles for selecting Q-Markers, brevilin A, arnicolide C, arnicolide D, and *Helenalin* possess pharmacological effects such as antitumor activity, indicating effectiveness. These components are present in *C. minima* at high levels and exhibit specificity compared to closely related species. Furthermore, there are currently accurate and reliable methods for their quantitative determination, ensuring measurability. However, other components such as caffeic acid, 3,5-di-O-caffeoylquinic acid, 4,5-di-O-caffeoylquinic acid, chlorogenic acid, and rutin are widely distributed in other medicinal materials, lacking specificity and therefore suitability as Q-Marker selections. Therefore, brevilin A, arnicolide C, arnicolide D, and *Helenalin* can serve as potential Q-Marker candidates for *C. minima*.

## 7. Conclusions

This review systematically consolidates the current knowledge on the diverse chemical constituents and multifaceted pharmacological activities of *C. minima*, underscoring its significant potential as a source of therapeutic agents. The plant produces a rich array of bioactive compounds, including volatile oils, flavonoids, and sesquiterpene lactones, which are responsible for its demonstrated anti-inflammatory, antitumor, and other pharmacological effects. Despite this promising foundation, our analysis reveals that the translation of these findings into standardized and safe clinical applications is impeded by several interconnected research gaps.

Substantial challenges remain across the board. Firstly, the chemical research is incomplete, with critical components like polysaccharides being poorly characterized, and the fundamental structure–activity relationships for most constituents are still unknown. Secondly, the understanding of pharmacological mechanisms is fragmented, particularly for volatile oils, and the complex synergistic interactions between compounds are largely unexplored. Finally, and most critically, the safety profile of C. minima is inadequately defined. It lacks comprehensive pharmacokinetic and toxicological studies, especially concerning its known gastrointestinal irritation and the potential toxicity of lipophilic components.

To bridge these gaps, a more systematic and integrated research strategy is imperative. Future efforts must prioritize the in-depth exploration of structure–activity relationships, the application of multi-omics and other advanced technologies to elucidate complex mechanisms, and rigorous safety assessments that include investigating traditional processing methods to mitigate adverse effects. Concurrently, establishing robust quality control standards, potentially using specific sesquiterpene lactones as quality markers, is essential for ensuring the consistency, efficacy, and safety of *C. minima*-based preparations. Addressing these priorities cohesively will be crucial for validating its traditional uses and unlocking its full potential in modern evidence-based medicine.

## Figures and Tables

**Table 1:** Chemical composition of volatile oils.

No.	Categories	Chemical Compound	Chemical Formula	Relative Content (%)	Reference
1	Monoterpene	Camphene	C_10_H_16_	0.08	[8]
2		Beta-pinene	C_10_H_16_	0.11	[8]
3		P-cymene	C_10_H_14_	0.24	[8]
4		(+)-Limonene	C_10_H_16_	0.27	[8]
5		γ-terpinene	C_10_H_16_	0.13	[8]
6		Myrcene	C_10_H_16_	0.32	[8]
7		1,4-Dimethyl-5-isopropylcyclopentene	C_10_H_18_	0.22	[8]
8	Oxygenated monoterpene	2,4,6-Trimethyl-3-cyclohexene-1-Carboxaldehyde	C_10_H_16_O	0.84	[7]
9		α-Phellandrene-8-ol	C_10_H_16_O	0.14	[7]
10		α-Terpineol	C_10_H_18_O	0.22	[7]
11		Bornyl acetate	C_12_H_20_O_2_	0.29	[7]
12		Thymol	C_10_H_14_O	5.69	[7]
13		Linalyl isobutyrate	C_14_H_24_O_2_	1.57	[7]
14		Geranyl isovalerate	C_15_H_26_O_2_	1.66	[7]
15		Cineole	C_10_H_18_O	1.53	[8]
16		Linalool	C_10_H_18_O	0.51	[7,8]
17		(E)-para-2-menthen-1-ol	C_10_H_18_O	0.11	[8]
18		Camphor	C_10_H_16_O	0.35	[7,8]
19		(−)-Verbenone	C_10_H_14_O	2.76	[8]
20		4-Methyl-1-isopropyl-3-cyclohexenol	C_10_H_18_O	0.78	[8]
21		Terpineol	C_10_H_18_O	0.22	[8]
22		Nerol	C_10_H_18_O	0.15	[7,8]
23		Trans-Chrysanthenyl acetate	C_12_H_18_O_2_	20.23	[7,8]
24		Carvacrol	C_10_H_14_O	0.52	[7,8]
25		Linalyl acetate	C_12_H_20_O_2_	2.20	[8]
26		Neo-Menthol	C_10_H_20_O	1.51	[7]
27	Sesquiterpene	α-Guaiene	C_15_H_24_	1.00	[7]
28		1,4,6-trimethyl-5,6-dihydronaphthalene	C_13_H_16_	0.14	[7]
29		α-Amorphene	C_15_H_24_	0.15	[7]
30		α-Copaene	C_15_H_24_	1.27	[7]
31		α-Gurjunene	C_15_H_24_	0.91	[7]
32		Sativen	C_15_H_24_	0.17	[7]
33		Allo-Aromadendrene	C_15_H_24_	1.20	[7]
34		α-Santalene	C_15_H_24_	0.87	[7]
35		α-Bergamotene	C_15_H_24_	0.23	[7]
36		Longifolene	C_15_H_24_	0.65	[7]
37		Humule[7,8]ne	C_15_H_24_	1.83	[7]
38		γ-Cadinene	C_15_H_24_	0.31	[7]
39		Aromadendrene	C_15_H_24_	11.47	[7]
40		β-Selinene	C_15_H_24_	0.18	[7]
41		β-Chamigrene	C_15_H_24_	0.89	[7]
42		α-Bisabolene	C_15_H_24_	0.21	[7]
43		Valencene	C_15_H_24_	0.57	[7]
44		1,1,6-trimethyl-1,2-dihydronaphthalene	C_13_H_16_	0.29	[7]
45		Isolongifolene	C_15_H_24_	1.09	[7,8]
46		1-Isopropyl-4,7-dimethyl-1,2,4a,5,8,8a-hexahydronaphthalene	C_15_H_24_	0.43	[8]
47		α-bulnesene	C_15_H_24_	0.71	[8]
48		Isocaryophyllene	C_15_H_24_	0.73	[8]
49		α-bergamotene	C_15_H_24_	1.87	[8]
50		β-Santalene	C_15_H_24_	2.29	[7,8]
51		β-caryophyllene	C_15_H_24_	5.26	[7,8]
52		β-Cedrene	C_15_H_24_	0.21	[8]
53		β-bisabolene	C_15_H_24_	0.55	[7,8]
54		β-cubebene	C_15_H_24_	0.11	[8]
55		Longicyclene	C_15_H_24_	0.59	[7]
56		1-Methyl-2,4-diisopropenylcyclohexane	C_13_H_22_	0.22	[8]
57	Oxygenated sesquiterpene	β-Caryophyllene oxide	C_15_H_24_O	1.03	[7]
58		α-Cadinol	C_15_H_26_O	0.23	[7]
59		Aromadendrene oxide	C_15_H_24_O	0.48	[7]
60		Cedrenol	C_15_H_24_O	0.47	[7]
61		Spathulenol	C_15_H_24_O	0.38	[7]
62		Cedr-8-en-13-ol	C_15_H_24_O	0.35	[7]
63		Muurolol	C_15_H_26_O	0.33	[7]
64		Globulol	C_15_H_26_O	0.53	[7]
65		Valerenal	C_15_H_22_O	0.56	[7]
66		Cedrol	C_15_H_26_O	0.25	[7]
67		Hexahydrofarnesyl acetone	C_18_H_36_O	1.28	[7]
68		D-Nerolidol	C_15_H_26_O	0.54	[7]
69		α-Bisabolol	C_15_H_26_O	0.22	[8]
70	Diterpenes	Kaur-16-ene	C_20_H_32_	0.53	[7]
71		Neophytadiene	C_20_H_38_	0.74	[7]
72	Aliphatic hydrocarbons	Pentadecane	C_15_H_32_	0.25	[7]
73	Oxygenated aliphatic	1-Nonanol	C_9_H_20_O	0.11	[8]
74		Methyl hexadecanoate	C_17_H_34_O_2_	0.59	[7]
75	Aromatic compounds	2,3,5,6-tetramethyl-Phenol	C_10_H_14_O	2.49	[7]
76		3-Ethyl-1,2,4,5-tetramethylbenzene	C_12_H_18_	1.05	[7]
77		2-isopropylphenol	C_9_H_12_O	0.44	[8]
78	Phenylbutazone	2-Allyl-6-methylphenol	C_10_H_120_	0.20	[7]
79		4-methyl-2-prop-2-enyl-phenol	C_10_H_12_O	0.38	[8]
80		4,7-dimethyl-3(2H)-Benzofuranone	C_10_H_10_O_2_	0.49	[7]
81	Others	Dibenzofuran	C_12_H_8_O	4.24	[7]
82		1,2,3,6-Tetramethylbicyclo [2.2.2]octa-2,5-diene	C_12_H_18_	2.79	[7,8]

**Table 2:** Chemical composition of terpenoids and their glycosides.

No.	Categories	Chemical Compound	Chemical Formula	References
1	Monoterpene	10-Isobutyryloxy-8,9-epoxythymol isobutyrate	C_18_H_24_O_5_	[10]
2		Thymohydroquinone-3-O-β-6′-acetyl-Glc	C_18_H_26_O_8_	[11]
3		Thymohydroquinone-6-O-β-6′-acetyl-Glc	C_18_H_26_O_8_	[11]
4		Zataroside-A	C_16_H_24_O_7_	[11]
5		Zataroside-B	C_16_H_24_O_7_	[11]
6		Thymol-3-O-β-glucoside	C_16_H_24_O_6_	[11,12]
7		(−)-Cis-chrysanthenol-O-β-D-glucopyranoside	C_16_H_26_O_6_	[11,13]
8		Thymoquinol 2-O-β-glucopyranoside	C_16_H_24_O_7_	[12]
9		Thymoquinol 5-O-β-glucopyranoside	C_16_H_24_O_7_	[12]
10		[2-(1-Hydroxypropan-2-yl)-4-methylphenol]	C_10_H_14_O_2_	[14]
11		10-Dihydroxy-9-isobutyryloxythymol	C_14_H_20_O_5_	[15,16]
12		8-Hydroxy-10-isobutyryloxy-9-(2-methylbutanoyl)	C_19_H_28_O_6_	[15]
13		Isobutyric acid 2-(2-hydroxy-4-methyl-phenyl)-3-isobutyryloxy-allyl ester	C_18_H_24_O_5_	[15]
14		9-Carboxylthymol	C_10_H_12_O_3_	[15]
15		9-Hydroxythymol	C_10_H_14_O_2_	[15,16]
16		8-Hydroxy-9,10-diisobutyryloxythymol	C_18_H_26_O_6_	[15,17,18]
17		8-Hydroxy-9-isobutyryloxy-10(2)-methylbutyryloxythymol	C_19_H_28_O_6_	[16]
18		E-p-coumaryl alcohol ethyl ether	C_11_H_14_O_2_	[16]
19		8,10-Dihydroxy-9(2)-methylbutyryloxythymol	C_15_H_22_O_5_	[18]
20		10-Hydroxy-8,9-dioxyisopropylidenethymol	C_13_H_18_O_4_	[18]
21	Sesquiterpene	Brevilin A	C_20_H_26_O_5_	[11,13] [15,17,19,20,21]
22		Arnicolide C	C_19_H_26_O_5_	[11,13,15,19,20,21]
23		Senecioylplenolin	C_20_H_26_O_5_	[22]
24		Arnicolide D	C_19_H_24_O_5_	[11,13,15,17,19,20,21,23]
25		Helenalin	C_15_H_18_O_4_	[11,14,24]
26		Tetrahydrohelenalin	C_15_H_22_O_4_	[22]
27		Tomentosin	C_15_H_20_O_3_	[22]
28		α-Cyperone	C_15_H_22_O	[25]
29		2-Methoxytetrahydrohelenalin	C_16_H_24_O_5_	[11]
30		Dihydrohelenalin	C_15_H_20_O_4_	[11,12]
31		2β-Hydroxyl-2,3-dihydrogen-6-O-angeloplenolin	C_20_H_28_O_6_	[14]
32		11,13-Dihydrohelenalin	C_15_H_20_O_4_	[14]
33		Microhenalin C	C_20_H_26_O_5_	[15,17,21]
34		Minimolide F	C_19_H_26_O_5_	[15,19,21,23]
35		2-β-(Isobutyryloxy)florilenalin	C_19_H_26_O_5_	[15]
36		(1S,2R,5R,6S,7R,8R,10R,11S)-4-Oxo-2β-butoxy-6α-angeloyloxypseudoguaia-8β,12-olide	C_24_H_36_O_6_	[17]
37		Centiplide A	C_24_H_36_O_6_	[17]
38		Cenminolide C	C_19_H_24_O_5_	[17]
39		Cenminolide A	C_20_H_26_O_5_	[17]
40		Minimolide B	C_22_H_32_O_6_	[23]
41		Arnicolide A	C_17_H_22_O_5_	[19]
42		Minimolide C	C_20_H_28_O_6_	[19,23]
43		Minimolide A	C_20_H_28_O_6_	[19,23]
44		Minimolide H	C_20_H_26_O_6_	[19,24]
45		Microhelenin C	C_20_H_26_O_5_	[19]
46		Minimolide E	C_20_H_26_O_5_	[19,23]
47		Minimolide D	C_20_H_28_O_5_	[19,23]
48		Arnicolide G	C_18_H_24_O_5_	[19]
49		Minimolide G	C_19_H_26_O_6_	[24,26]
50		Plenolin	C_15_H_20_O_4_	[23]
51		Pulchellin-2α-O-isovalerate	C_21_H_32_O_5_	[23]
52		Florilenalin-2α-O-isovalerate	C_20_H_28_O_5_	[23]
53		Florilenalin-2α-O-angelate	C_20_H_26_O_5_	[23]
54		Minimaoside B	C_21_H_34_O_9_	[27]
55		4,5b-Dihydroxy-2β-(isobutyryloxy)-10βH-guai-11(13)-en-12,8β-olide	C_19_H_28_O_6_	[28]
56		4-Hydroxy-1βH-guaia-9,11(13)-dien-12,8α-olide	C_15_H_20_O_3_	[28]
57		2β-(Isobutyryloxy)florilenalin	C_19_H_26_O_5_	[28]
58		Pulchellin-2α-O-tiglate	C_20_H_28_O_5_	[28]
59		Florilenalin-2α-O-tiglate	C_20_H_26_O_5_	[28]
60	Triterpene	Arnidiol	C_30_H_50_O_2_	[29]
61		Lupeol	C_30_H_50_O	[29]
62		Ursane-20(30)-en-3β,16β,21α-triol	C_30_H_50_O_3_	[30]
63		Lupeol acetate	C_32_H_52_O_2_	[16]
64		Friedelin	C_30_H_50_O	[16]
65		Guaiacin D	C_46_H_72_O_16_	[31]
66		Nudicaucin A	C_46_H_72_O_17_	[31]
67		Lupenone	C_30_H_48_O	[16]
68		20-Oxo-30-nortaraxastan-3β-yl acetate	C_31_H_50_O_3_	[29]
69		3β-Acetoxytaraxaster-20-en-30-al	C_32_H_50_O_3_	[29]
70		3β-Hydroxytaraxaster-20-en-30-al	C_30_H_48_O_2_	[29]
71		Faradiol	C_30_H_50_O_2_	[29]
72		Taraxast-20-ene-3β,30-diol	C_30_H_50_O_2_	[29]
73		Taraxast-20-ene-3β,30-diol	C_30_H_46_O_2_	[29]
74		18α-Oleana-12-en-3,11-dione	C_30_H_50_O_2_	[29]
75		Maniladiol	C_30_H_48_O_2_	[29]
76		3β-Hydroxy-oleana-12-en-11-one	C_30_H_50_O_2_	[29]
77		Coflodiol	C_30_H_50_O_2_	[29]
78		3β,16β-Hydroxy-lupine diol	C_30_H_50_O_2_	[29]
79		Lup-20(29)-ene-3b,16b-diol	C_30_H_52_O_2_	[29]
80		Garcinielliptone Q	C_30_H_50_	[16]
81		Taraxasterol acetate	C_32_H_52_O_2_	[16]
82		Pesudotaraxasterol acetate	C_32_H_52_O_2_	[16,29]
83		Taraxasterone	C_30_H_48_O	[16]
84		3β,21β-Dihydroxy-20(30)-en-taraxastane	C_30_H_50_O_2_	[29]
85		Taraxasterol	C_30_H_50_O	[14,16,29]

**Table 3:** Chemical composition of flavonoids and their glycosides.

No.	Chemical Compound	Chemical Formula	References
1	Quercetin-3-methyl-ether	C_16_H_12_O_7_	[2]
2	Quercetin 3,3′-dimethyl ether	C_17_H_14_O_7_	[2]
3	Apigenin	C_15_H_10_O_5_	[11]
4	Nobiletin	C_21_H_22_O_8_	[33]
5	Luteolin	C_15_H_10_O_6_	[11]
6	Tricin	C_17_H_14_O_7_	[11,13]
7	2-O-Methylquercetin	C_16_H_12_O_7_	[11]
8	Quercetin	C_15_H_10_O_7_	[11,12,34]
9	Rutin	C_27_H_30_O_16_	[11,20]
10	Kaempferol-3-O-α-L-rhamnopyranosyl-(1→6)-β-D-glucopyrano side	C_27_H_30_O_15_	[11]
11	3-O-Methylquercetin	C_16_H_12_O_7_	[17,34]
12	(2R,3R)-(+)-7,4′-Di-O-methyldihydrokaempferol	C_17_H_16_O_6_	[34]
13	Iristectorin A	C_23_H_24_O_12_	[34]
14	Isocutellarein 7-methyl ether	C_16_H_12_O_6_	[34]
15	Hispidulin	C_16_H_12_O_6_	[34]
16	Casticin	C_19_H_18_O_8_	[35]
17	Artemitin	C_20_H_20_O_8_	[35]
18	Chrysosplenol D	C_18_H_16_O_8_	[36]
19	Luteolin-7-O-glucoside	C_21_H_20_O_11_	[31]
20	Quercetin-3-O-β-D-glucoside	C_21_H_20_O_12_	[31]
21	Apigenin-7-O-β-D-glucuronide	C_21_H_18_O_11_	[31]
22	Kaempferol-3-O-rutinoside	C_27_H_30_O_15_	[20]
23	Isorhamnetin	C_16_H_12_O_7_	[20]

**Table 4:** Chemical composition of sterols.

No.	Chemical Compound	Chemical Formula	References
1	β-sitosterol	C_30_H_52_O	[11,12,16,37]
2	Stigmasterol	C_29_H_48_O	[11,12,17]
3	Stigmasterol-3-O-β-D-glucopyranoside	C_35_H_58_O_6_	[14]
4	Spinasterol	C_29_H_48_O	[37]
5	[5α,8α-epidioxy-(22E,20S,24R)-ergosta-6,22-dien-3β-ol]	C_28_H_44_O_3_	[37]
6	β-Sitosterylpalmitate	C_35_H_60_O_6_	[14]

**Table 5:** Chemical composition of organic acids.

No.	Chemical Compound	Chemical Formula	References
1	Ethyl caffeate	C_11_H_12_O_4_	[11,12]
2	Methy 3,5-Di-O-caffeoylquinate	C_26_H_26_O_12_	[11,13]
3	3,5-Di-O-caffeoylquinicacid	C_25_H_24_O_12_	[11,13]
4	DL-leucine	C_6_H_13_NO_2_	[11,13]
5	Dl-Phenylalanine	C_9_H_11_NO_2_	[11,13]
6	4-Amino-2-oxo-3H-chromene-4-carboxylic acid	C_10_H_9_NO_4_	[11,13]
7	Benzoic acid	C_7_H_6_O_2_	[35,36]
8	Pentadecanoic acid	C_15_H_30_O_2_	[39,40]
9	Stearic Acid	C_18_H_36_O_2_	[39,40]
10	Uracil	C_4_H_4_N_2_O_2_	[39,40]
11	N-hexadecanoic acid	C_16_H_32_O_2_	[39]
12	Adenine	C_5_H_5_N_5_	[40]
13	Uridine	C_9_H_12_N_2_O_6_	[40]
14	Chlorogenic acid	C_16_H_18_O_9_	[20,31]
15	Protocatechuicacid	C_7_H_6_O_4_	[15]
16	Paraben	C_7_H_6_O_3_	[31]
17	Salicylic acid	C_7_H_6_O_3_	[31]
18	Sinapic acid	C_11_H_12_O_5_	[31]
19	Methyl sinapate	C_12_H_14_O_5_	[31]
20	Ferulic acid	C_10_H_10_O_4_	[31]
21	Methyl caffeate acid	C_10_H_10_O_4_	[31]
22	Syringic acid	C_9_H_10_O_5_	[31]
23	Vanillic acid	C_8_H_8_O_4_	[16]
24	Cryptochlorogenic acid	C_16_H_18_O_9_	[20]
25	Caffeic acid	C_9_H_8_O_4_	[20]
26	Isochlorogenic acid B	C_25_H_24_O_12_	[20]
27	Isochlorogenic acid A	C_25_H_24_O_12_	[20]
28	Isochlorogenic acid C	C_25_H_24_O_12_	[20]
29	3-O-caffeoyl-α-glueopyranose	C_15_H_18_O_9_	[34]
30	3-O-caffeoyl-β-glucopyranose	C_15_H_18_O_9_	[34]
31	4,5-Di-O-caffeoylquinic acid methyl ester	C_26_H_26_O_12_	[31]
